# Alphavirus Particles Can Assemble with an Alternate Triangulation Number

**DOI:** 10.3390/v14122650

**Published:** 2022-11-27

**Authors:** Jason T. Kaelber, David Chmielewski, Wah Chiu, Albert J. Auguste

**Affiliations:** 1 Institute for Quantitative Biomedicine, Rutgers University, Piscataway, NJ 08854, USA; 2 Biophysics Graduate Program, Department of Bioengineering, Stanford University, Stanford, CA 94305, USA; 3 Biophysics Graduate Program, Department of Microbiology and Immunology, Stanford University, Stanford, CA 94305, USA; 4 Division of CryoEM and Bioimaging, SSRL, SLAC National Accelerator Laboratory, Menlo Park, CA 94025, USA; 5 Department of Entomology, College of Agriculture and Life Sciences, Fralin Life Science Institute, Virginia Polytechnic Institute and State University, Blacksburg, VA 24061, USA; 6 Center for Emerging, Zoonotic, and Arthropod-borne Pathogens, Virginia Polytechnic Institute and State University, Blacksburg, VA 24061, USA

**Keywords:** alphavirus, quasi-equivalence, triangulation number, *Togaviridae*, virus assembly, cryo-electron microscopy

## Abstract

Alphaviruses are spherical, enveloped RNA viruses primarily transmitted by mosquitoes, and cause significant arthritogenic and neurotropic disease in humans and livestock. Previous reports have shown that—in contrast to prototypical icosahedral viruses—alphaviruses incorporate frequent defects, and these may serve important functions in the viral life cycle. We confirm the genus-wide pleomorphism in live viral particles and extend our understanding of alphavirus assembly through the discovery of an alternate architecture of Eastern equine encephalitis virus (EEEV) particles. The alternate *T* = 3 icosahedral architecture differs in triangulation number from the classic *T* = 4 icosahedral organization that typifies alphaviruses, but the alternate architecture maintains the quasi-equivalence relationship of asymmetric units. The fusion spike glycoproteins are more loosely apposed in the *T* = 3 form with corresponding changes in the underlying capsid protein lattice. This alternate architecture could potentially be exploited in engineering alphavirus-based particles for delivery of alphaviral or other RNA.

## 1. Introduction

Viruses typically form icosahedral shells or helical tubes by repeating a capsomere (made up of one or a few protein subunits) in a structured design to protect their genome and form an active virion [1]. Each copy of the capsomere has the same shape and they tile [2] across the shell in a regular pattern. The geometric requirements of this tessellation constrain how many capsomeres can be present and how they are arranged in the shell [1,2]. For viruses obeying the classical Caspar-Klug quasi-equivalent arrangement of subunits, their architecture is described by the triangulation number *T*, and they have 60 × *T* capsomeres.

The *Alphavirus* genus (family *Togaviridae*) contains arthropod-transmitted pathogens responsible for near-global epidemics in humans and livestock [3]. Arthritogenic alphaviruses such as Chikungunya virus (CHIKV) and Mayaro virus (MAYV) cause debilitating and often persistent polyarthritis, while neurotropic alphaviruses such as Eastern equine encephalitis virus (EEEV) and Venezuelan equine encephalitis virus (VEEV) have high case fatality rates [4]. Spherical alphavirus particles possess icosahedral *T* = 4 symmetry with 80 envelope glycoprotein trimers on the surface, each formed by three E1/E2 heterodimers. An inner nucleocapsid core containing the genome is formed by 240 capsid proteins (Cps) also arranged in *T* = 4 icosahedral symmetry.

During alphavirus infection, Cp auto-proteolytic cleavage from the structural polypeptide leads to genomic association and cytosolic NC assembly. Alphavirus particles assemble and bud at the plasma membrane, where envelope glycoprotein complexes transported through the secretory system bind and enwrap NCs through E2:Cp interactions. A conserved E2 sequence in the cytoplasmic tail binds to a hydrophobic Cp pocket, linking two protein layers across the membrane. Interestingly, Cps that fail to assemble into cytoplasmic NCs can form *T* = 4 icosahedral virions during budding, although production of such particles is inefficient [5,6]. In addition, anti-CHIKV antibodies that disrupt spike-spike interactions were shown to prevent virus budding, suggesting lateral envelope interactions drive the assembly/budding of released virions. Within released virions and in vitro-assembled cores, previous biochemical and 2D structural data indicated that Ross River virus (RRV) particles do not form complete icosahedral NC shells and incorporate defects [7]. A capsid mutant of Sindbis virus can form particles of variable, cargo-dependent size, underscoring the promiscuity of the alphavirus assembly pathway [8].

To analyze virion structures produced by alphavirus assembly/budding, we purified and imaged four different viruses from the genus. Alphaviruses are highly infectious agents and several neurotropic alphaviruses (WEEV, VEEV, EEEV) are capable of causing serious and potentially fatal infections of the central nervous system. Therefore, wild-type (WT) infectious neurotropic alphaviruses are classified as priority pathogens and Biosafety level 3 (BSL3) agents requiring increased biocontainment facilities. Wild-type arthritogenic alphaviruses such as CHIKV are classified as BSL2+ and typically require similar biocontainment and biosecurity practices. Due to the absence of these facilities and the inability to fully enclose the specimen preparation devices and electron microscopes in our facility, it was necessary to handle viruses at BSL2 or below. Therefore, we used a chimeric virus based on the insect-specific Eilat virus (EILV) that can be handled under BSL1 laboratory conditions [9]. The EILV/EEEV virus chimera contains structural proteins from EEEV (strain EEEV-FL 93-939) and nonstructural proteins from Eilat virus [10]. Similarly, an analogous chimera using chikungunya virus (CHIKV) does not differ structurally from wild-type CHIKV virus [11].

Here, we analyze the particle morphologies and 3D structures of neurotropic and arthritogenic alphaviruses and describe a wide range of assembly outcomes. Compared to a control virus known for its structural monodispersity, alphaviruses are more likely to exhibit pleomorphism including large, multi-cored particles. Furthermore, we identify a stable *T* = 3 viral particle as a minor component of a purified alphavirus preparation. Structural heterogeneities are an inherent feature of alphavirus particles and deviations from the theoretically predicted *T* = 4 perfectly icosahedral structure are plentiful.

## 2. Materials and Methods

### 2.1. Purification and Electron Microscopy of Alphavirus Particle Morphologies

EILV/EEEV, EILV/CHIKV, and EILV/VEEV chimeric alphaviruses were generated by replacing the structural protein open reading frame (str ORF) of EILV with that of wild-type EEEV, CHIKV and VEEV, respectively, as previously described [10,11]. Briefly, the region of the genome corresponding to the 26S subgenomic RNA (which contains the structural polyprotein but not the subgenomic promoter or 3′ noncoding regions of the genome) was amplified by PCR, digested with restriction enzymes, and ligated into pEILV [12] to give rise to a full-length viral cDNA clone whose sequence was confirmed by Sanger sequencing. The PCR fragment was amplified from reverse-transcribed viral DNA in the case of CHIKV (strain CHIKV-99659) and from infectious clones in the cases of VEEV (strain TC-83) or EEEV (strain FL93-939). Each virus construct was independently amplified by infection of cultured C7/10 mosquito cells at a multiplicity (MOI) of 0.1 PFU/cell, and at 48 h post-infection (HPI), the supernatants were harvested and clarified by centrifugation for 10 min at 3000× *g*. To concentrate virus, clarified supernatants were mixed with polyethylene glycol (PEG) 8000 and NaCl to 7% and 2.3% (wt/vol), incubated overnight at 4 °C, and the precipitate was pelleted by centrifugation for 30 min at 3100× *g*. Pellets were then resuspended in PBS and titrated by C7/10 plaque assay as described below. Resuspended virus was overlaid onto a 20–70% continuous sucrose gradient and separated from contaminants by ultracentrifugation for 1.5 h at 210,000× *g*. The virus band was collected and applied to a 100-kDa Amicon filter (Millipore, Billerica, MA). Residual sucrose was then removed by 5 washes with PBS. The EILV/X virions (where X refers to EEEV, CHIKV or VEEV str ORF) in Tris/EDTA/NaCl (TEN) buffer (0.05 M Tris-HCl [pH 7.4], 0.1 M NaCl, 0.001 M EDTA) were diluted with an equal volume of TEN buffer to reduce particle concentration. Three μL of the diluted virion solution was applied to a Quantifoil grid (mesh size 200) and blotted with an FEI Vitrobot Mark IV for 2 s at 100% relative humidity and room temperature before plunge freezing. Preliminary imaging on a JEM-2010F cryomicroscope was used for initial model generation in EMAN2 [13]. Final imaging was performed in a JEM-3200FSC cryomicroscope with Omega-type energy filter. Micrographs of EILV/CHIKV and EILV/VEEV were recorded at 1.96 Å/pix on a DE-20 direct electron detector. 60 electrons per Å^2^ were captured. Damage compensation and motion correction were performed as previously described [14]. Micrographs of EILV/EEEV were collected in the JEM-3200FSC cryomicroscope with K2 Summit electron detector operated in counted mode at 1.71 Å/pix.

VEEV-TC83 vaccine strain virus was grown in baby hamster kidney cells, prepared to 80–90% confluence and inoculated with virus at a multiplicity of 0.1 PFU/cell. Infected cells were incubated at 37 °C for 2 days until cytopathic effects appeared; then the supernatant was clarified by centrifugation for 5–10 min at 1000–2000× *g* to remove cellular debris. The virus was concentrated by precipitation with 7% polyethylene glycol 6000 and 2.3% NaCl at 4 °C for >4 h. Then, the virus was centrifuged at ≥2500× *g* for 30 min and was gently resuspended in 1–2 mL TEN buffer (0.05 M Tris–HCl, pH 7.4, 0.1 M NaCl and 0.001 M EDTA). The virus suspension was purified by centrifugation through a 20–70% sucrose/TEN continuous gradient for 60 min at 35,000× *g*. The virus band was harvested using a plastic Pasteur pipette and centrifuged 3× *g* through Amicon 100 kDa filter (Ultra-4 Cat. No. UFC810024), resuspending each time to maximum load volume with TEN. The purified virus was harvested in the minimal remaining volume after final centrifugation (ca. 50–100 μL). Cryo-EM grids were prepared and micrographs collected on the JEM 3200FSC cryo-transmission electron microscope with Omega-type energy filter at a detector magnification of 141,110× (1.07 Å/pixel sampling rate) using a Gatan 4K × 4K CCD camera.

Adeno-associated virus serotype 8 (AAV8) was repurified in phosphate-buffered saline (PBS) as previously described [15]. Diluted virion solution was applied to a glow-discharged Quantifoil grid (mesh size 200) with a continuous carbon overlay with thickness between 2 and 3 nm, as measured by quartz oscillation frequency in the Leica ACE600 used to deposit the carbon on cleaved mica by e-beam deposition. Imaging was performed in a Talos Arctica cryomicroscope with Gatan Bioquantum energy filter with K2 Summit electron detector operated in counted mode at 1.04 Å/pix.

The datasets of micrographs of EILV/VEEV [10], EILV/CHIKV [11], and VEEV-TC83 [16] have been previously published while the datasets of EILV/EEEV and AAV8 have not been published hitherto. Metadata traceability for all alphavirus datasets was effected via EMEN2 lab notebook/database [17].

### 2.2. EEEV Cryo-EM Data Processing

Particles were boxed using EMAN2.1 and then motion/damage-corrected using the script DE_process_frames.py [14]. Initial, iterative image alignment and reconstruction was performed in the package MPSA [18], which aligns images by the method of common lines. This achieved pseudoconvergence in 5 iterations. This result was not utilized in further refinements. To investigate particle heterogeneity, all 23,332 accepted particles were subjected to two-class ab initio refinement in cryoSPARC v1 [19] without the use of any input model, but with enforcement of icosahedral symmetry. One of the resulting classes exhibited *T* = 4 quasi-equivalence and the other *T* = 3. To improve the resolution of these classes, each was refined independently. The particles belonging to the *T* = 4 class were iteratively aligned by projection-matching and averaged starting from the *T* = 4 ab initio model (implemented in cryoSPARC as “homogeneous refinement”), and (separately) the same was done for the *T* = 3 class. Refinement improved map resolution (to ~10 Å in the case of the *T* = 3 subset) but did not alter the triangulation number. The less-populous *T* = 3 class contained 1540 out of 23,332 particles. To confirm that the difference in triangulation number was inherent to the particle subset and not a reconstruction artifact of the package, the particle subsets were isolated for reprocessing in EMAN2.1 [20]. CryoSPARC particle lists in csv format were parsed using *pyem* scripts (Daniel Asarnow; https://github.com/asarnow/pyem (accessed on 29 April 2020)) and then converted onward to EMAN format. The list of putative *T* = 3 particles was refined with icosahedral symmetry using as an initial model the cryoSPARC *T* = 3 reconstructed volume phase-randomized to 30 Å; the resulting map at 13.2 Å exhibited *T* = 3 quasi-equivalence. That list of particles was also refined in the same way against the cryoSPARC *T* = 4 reconstructed volume to 13.4 Å and the resulting map exhibited *T* = 3 quasi-equivalence. The list of putative *T* = 4 particles was refined against cryoSPARC *T* = 3 or *T* = 4 volumes and yielded, in either case, a map with *T* = 4 quasi-equivalence at subnanometer resolution.

## 3. Results

### 3.1. Purified Alphavirus Particles Are Structurally Heterogeneous

**Figure 1:**
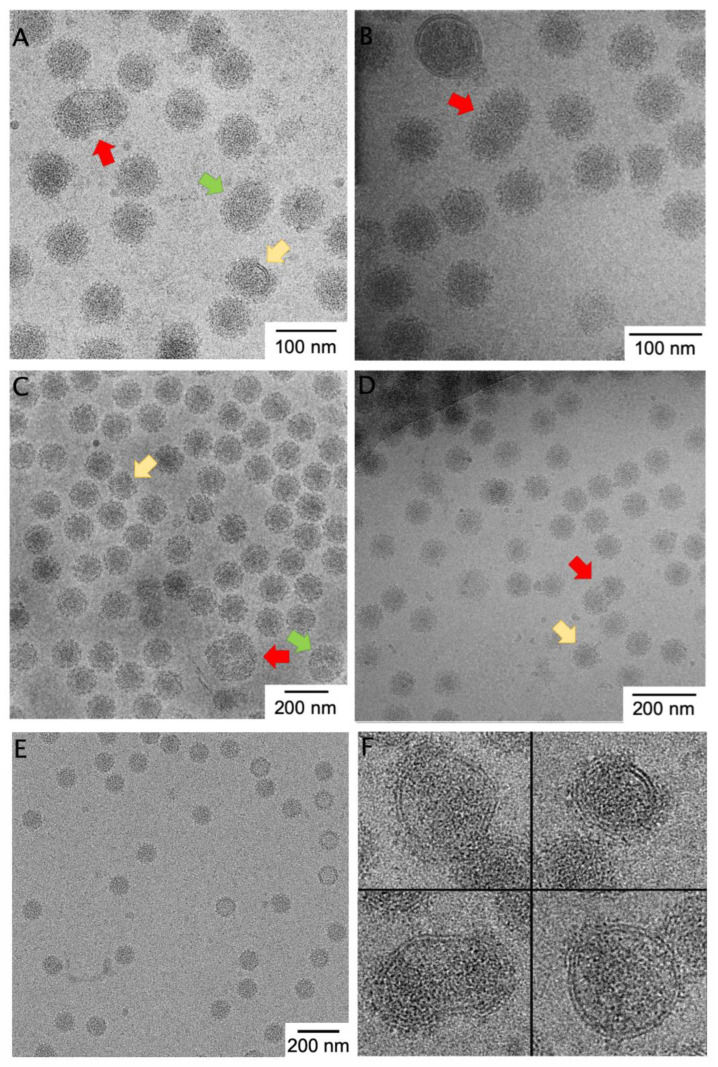
Micrographs of heterogeneous alphavirus preparations. Representative cryo-EM images of (**A**) EILV/EEEV, (**B**) VEEV TC-83, (**C**) EILV/CHIKV, and (**D**) EILV/VEEV purified in the same laboratory by comparable methods, as well as (**E**) the control virus AAV8. Multi-cored particles (red arrow)— particles with two or more NCs inside the viral envelope—were frequently observed in each alphaviral preparation but not in AAV8. Particles bigger (green arrow) and smaller (yellow arrow) than normal were also observed. (**F**) Selected pleomorphic particles of EILV/EEEV illustrate variable spike coverage and nucleocapsid defects which are sometimes, but not always, associated with multiple cores per particle.

To assess the pleomorphism in alphaviruses generally, we compared images of four alphaviruses. Taking advantage of the isogenic Eilat virus backbone and C6/36 expression system, we can compare micrographs of purified EILV/EEEV to EILV/CHIKV and EILV/VEEV all prepared by the same experimenter and using the same protocol (Figure 1). We also compare the non-chimeric attenuated TC-83 vaccine strain of VEEV (Figure 1B) [16], as well as a non-alphavirus control (Figure 1E). The control, AAV8, is a *T* = 1 non-enveloped ssDNA virus with high environmental stability. We randomly selected a number of micrographs, excluded micrographs in which particle morphology is hard to detect by eye, and then counted the number of particles that appeared normal, abnormally small (Figure 1, yellow arrows), abnormally large (green arrows), or multi-cored (i.e., having more than one core or partial core or having a dumbbell-shaped core inside the envelope; red arrows). Distinguishing larger or smaller particles was difficult because large changes in particle volume yield small changes in particle radius due to the cubic relationship, and because even normal-sized particles apparently contain discontinuities or imperfections in the protein lattice. However, multi-cored particles are easily identifiable (Figure 1, red arrows). Roughly 3% of viral particles were multi-cored in EILV/EEEV, EILV/CHIKV, and wild-type VEEV TC-83, while 1% of particles were multi-cored in EILV/VEEV (Table 1; Figure 1A–D). Multi-cored particles were stable for 120 weeks in solution (data not shown). By contrast, a control non-enveloped virus, AAV8, showed zero geminate particles and only 0.5% particles of abnormal diameter (Table 1; Figure 1E). From the prevalence and temporal stability of large, pleomorphic, multi-cored particles across alphaviruses, we conclude that such particles are likely present in vivo and reflect the frequent deviations from icosahedrality/quasi-equivalence in this genus.

Architecturally, cores within multi-cored particles had a circular or elliptical shape in projection and the particles contained areas of low density (no RNA) between cores within the same envelope. One could envision that the cores are “beads on a string” in a multi-plet configuration, where the ssRNA connecting neighboring cores is too small to observe. However, assuming a constant RNA:volume ratio within cores, a four-cored particle with just one RNA would encapsidate an RNA of over 40 kb in length, and such RNAs are exceedingly rare in the cytoplasm. Thus, we infer that a multi-cored particle contains multiple RNA molecules, each within its own nucleocapsid shell. This differs from typical cargo-driven T-number switching in viruses and may have a different etiology than the small particles discussed in the next section. Plausible non-RNA-driven mechanism for multi-cored particle formation include the presence of kinetic traps in budding or imbalance in glycoprotein/capsid ratio resulting in “starved” assembly [21].

We observed (Figure 1F) alphaviral particles that were incompletely covered with glycoprotein spikes (i.e., they contained patches of naked membrane) as well as particles with incomplete nucleocapsids, as evinced by smaller, non-spherical nucleocapsid morphologies. Nucleocapsid was not touching the membrane in areas where no glycoprotein coated the membrane, as we might expect a priori because Cp anchoring to the membrane is via the E1/E2 endodomains.

### 3.2. Two Icosahedral Architectures of Eastern Equine Encephalitis Virus

**Figure 2:**
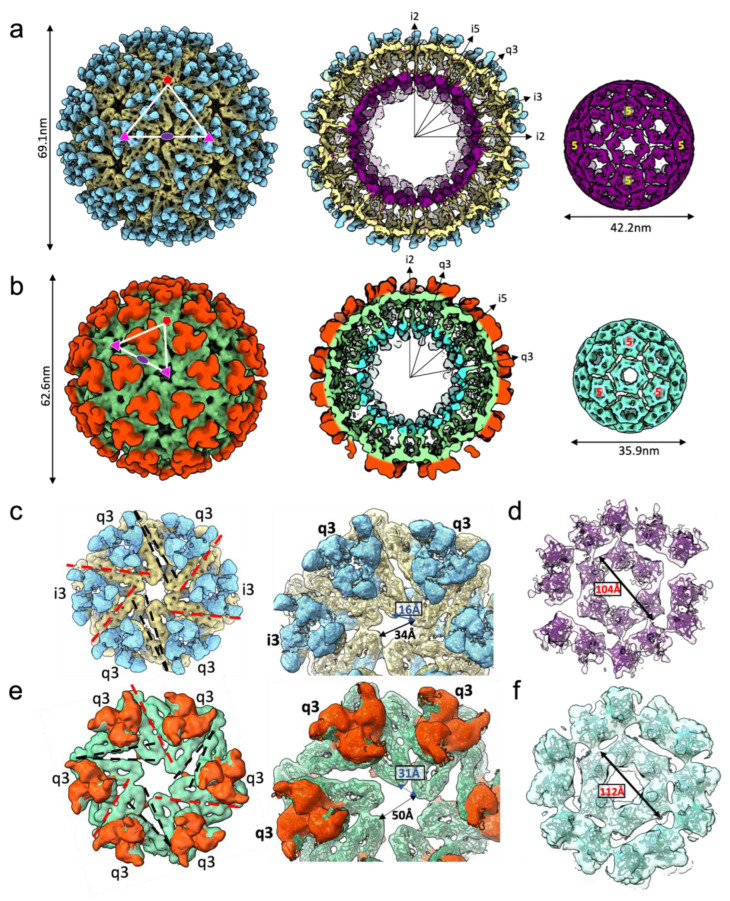
Cryo-EM structures of two icosahedral EEEV particles. Single-particle cryo-EM structures of EEEV (**a**) *T* = 4 and (**b**) *T* = 3 icosahedral particles, with radial-colored density map, half-cut representation and extracted NC density displayed left-to-right. 5-fold (red pentagon), 3-fold (pink triangle), and 2-fold (purple oval) symmetry axes labeled. (**c**) *T* = 4 glycoprotein hexamer density computationally extracted from the map (E2: blue, E1: khaki) with a zoom-in view on the right. Type I (extensive lateral contacts-red) and type II (limited angular contact-black) interfaces are labeled within the hexamer assembly. (**d**) *T =* 4 NC density (purple) around the hexamer assembly displayed with a rigid-fit of an atomic model of Cp C-terminal domain. (**e**) *T =* 3 glycoprotein hexamer density (E2-orange, E1-green) with a zoom-in view on the right and (**f**) *T =* 3 NC density (aqua) around hexamer with interfaces and distances labeled. PDB model 6MX4 rigid fit into density maps (**c**–**f**).

We investigated the structure of the subset of EILV/EEEV particles that appeared, by eye, to have normal morphology (Figure 1A). All particles were refined (with icosahedral symmetry enforced) to 7.5 Å resolution using the method of common lines as implemented in the software package MPSA [18], resulting in a *T* = 4 icosahedral reconstruction consistent with a published structure of EEEV [22] (Figure 2A; EMD-28780 main map). We suspected compositional heterogeneity might be limiting the achievable resolution, so we performed multi-model ab initio refinement in cryoSPARC v1 [19] to sort particles into classes; after, we refined each class independently by traditional projection-matching. Two structures with typical alphavirus features were obtained. Surprisingly, while the majority structure had the *T* = 4 architecture that is standard for all alphaviruses, a minority subset of particles yielded a *T* = 3 structure (Figure 2B; EMD-28780 additional map).

To exclude algorithmic bias, we also refined the two classes of particles in EMAN2 and obtained the same result. Then, to rule out initial model bias as an explanation for the observed *T* = 3 architecture, we iteratively refined particles from the *T* = 3 class starting from a *T* = 4 initial model. The refinement converged to a *T* = 3 map despite being biased towards *T* = 4. In the reciprocal experiment, particles from the *T* = 4 class refined from a *T* = 3 initial model also reproduced a *T* = 4 map. Excluding the two most likely pathologies in computational analysis, bolsters our confidence that the *T* = 3 architecture is present as a subpopulation of the purified EILV/EEEV virions. Approximately 7% of particles were sorted into the *T* = 3 class and were further refined to produce a reconstruction at ~10 Å resolution.

The *T* = 3 particle is approximately 34% smaller in volume (3.9 × 10^4^ nm^3^) than the *T* = 4 particle (5.2 × 10^4^ nm^3^) and contains 180 E1/E2/Cp subunits compared to 240 in the *T* = 4 virion (the charged surface therefore remains proportional to the volume). To achieve a tighter radius of curvature, structural interactions within the *T* = 3 spike shell and core must be different than those in the *T* = 4 particle. We then rigid-fit the atomic model for EEEV E1•E2•Cp subunit (PDB:6MX4) into the *T* = 4 and *T* = 3 reconstructions to analyze spike-spike interactions in the outer glycoprotein shell. Organization of spike trimers in the penton was similar between *T* = 4 and *T* = 3 particles. However, in the *T* = 4 particle, the E1 molecules forming the hexamer assemblies were more closely packed, containing four antiparallel, flat interfaces (q3:i3 trimers, type I) with extensive contacts, and two less extensive, angled interfaces (q3:q3, type II) with a larger gap between neighboring spikes (Figure 2C). In comparison, spikes forming the hexamer in the *T* = 3 virion were more loosely arranged and spaced further apart, with three flat, extensive interfaces (two q3:i3, one q3:q3) and three angled interfaces (two q3:i3, one q3:q3) (Figure 2E). This loose organization allowed for a higher curvature of the hexamer that facilitates the smaller diameter of the *T* = 3 icosahedron. Cps within the NC below were linked to spikes by interactions with the E2 C-terminal tails and arranged to form correlating *T* = 4 and *T* = 3 lattices, with the *T* = 3 NC hexamer again more loosely packed (Figure 2E,F). This suggests both the icosahedral spike and Cp lattices are pliable and able to accommodate different curved membrane geometries with quasi-equivalent interactions.

The interior volume that could be filled by RNA is 1.8 times larger for the native *T* = 4 architecture than for *T* = 3. However, alphavirus genomes are loosely packed: the native *T* = 4 virion containing a genome of 11.7 kb nt would have a Matthews coefficient (V_m_) greater than 5 Å^3^/Da, which is much higher than, for example, ssRNA picornaviruses and bromoviruses [23]. Therefore, it is plausible that the *T* = 3 virion could contain a complete genome with V_m_ = 3.1 Å^3^/Da. Alternatively, *T* = 3 particles could package a smaller RNA, such as the 26S subgenomic RNA of about 4.2kb, at even lower pressure.

## 4. Discussion

The stability of *T* = 3 virions, the existence of defects in the icosahedral organization of envelope glycoproteins and nucleocapsid cores (Wang et al., 2015), and the heterogeneity in alphavirus morphology all underscore that alphavirus particles are best understood as “mostly icosahedral” viruses. Because virion metastability is essential to viral survival, each virus has evolved to balance order and disorder of the virion structure. As compared to other types of symmetric viruses, alphaviruses are more pleomorphic and incorporate more defects. However, it remains unclear how or if these heterogeneities offer advantages during the virus life cycle.

Despite the outer protein shell, when alphavirus particles bud they form pleomorphic virions with various sizes and morphologies. We attribute the presence of multi-cored particles (containing spikes on the envelope surface and 2–5 nucleocapsid cores) to assembly events rather than post-budding fusion of released particles for two reasons. First, virus stored at 4° for almost two years showed no increase in multi-cored particle prevalence [10]. Second, we previously showed that NC cores docked closely at the plasma membrane could result in sharing of spikes across neighboring particle buds, forming what appears to be early intermediates of the multi-cored particles [24]. This can happen especially during infection with high MOI, where NCs are presumably delivered at high local concentration to budding sites. Such multi-cored particles, if endocytosed via spike-mediated receptor interactions, would result in high local multiplicity of infection (MOI) during entry. High MOI usually promotes virus infection by saturating host restriction factors [25,26], and contributes to modulation of viral pathogenesis by maintaining genetic diversity [27,28]. Alphavirus virions enveloping multiple cores is reminiscent of certain non-enveloped viruses that are packaged into enveloped microvesicles for dissemination, and are associated with a higher infectivity [29]. Future functional studies of these multi-cored alphaviruses, both in vitro and in vivo, are warranted. For example, does the producer cell species (e.g., *Aedes albopictus* vs. *Homo sapiens*) or cell type impact the relative abundance of *T* = 3 and *T* = 4 particles? Additionally, as cryoEM methods continue to improve, per-particle analysis (potentially by tomography) should reveal more insights into the provenance and structure of the *T* = 3 particles.

Alternate triangulation numbers have been observed for in vitro systems based on cowpea chlorotic mottle virus [30], brome mosaic virus [31], SV40 [32], and others. These correspond to “dead-end” assembly events reflecting the essential promiscuity of inter-subunit interactions. The best-characterized systems of *T*-number switching are related to cargo encapsulation. It could be that *T* = 3 virions are not formed in natural EEEV infections: although EILV/EEEV does not differ from wild-type EEEV in the sequence of its structural proteins it may produce RNA differently; it is conceivable that EILV non-structural proteins interact differently with EEEV structural proteins or RNA cis-acting elements differently than isogenic non-structural proteins would, and this could affect RNA production (or another step leading to proper virion assembly). Regardless, we have established that unmutated EEEV structural proteins can form RNA-containing *T* = 3 virions under some conditions of cell infection; their properly folded spikes indicate that they can likely fuse with human cells and deliver RNA—although this has not been experimentally confirmed. Further work is necessary to determine what RNA species is packaged into *T* = 3 virions and whether such particles relevant to alphavirus infection in vivo.

Assembly of both *T* = 4 and *T* = 3 virions in the same preparation raises the question of what determines the achieved icosahedral architecture of a released particle. It is conceivable that packaging of smaller RNA species such as 26S RNA could result in a smaller RNA-Cp NC intermediate in accordance with the proposal that NCs assemble as Cps neutralize the negative RNA charge [33]. Based on recent reports of CHIKV assembly/budding in situ, a smaller RNA/Cp assembly would serve as a rough scaffold that indirectly guides self-assembly of a smaller icosahedral spike lattice than the potentially larger cytoplasmic NC intermediate packaging the 49S genomic RNA [24]. When Aura virus is propagated in the C6/36 cells (the same cell line we used to propagate EILV/EEEV), 52% of the packaged RNA molecules were 26S RNA [34]. Negatively-stained Aura virus contained two classes of particles with diameters comparable to those observed here (62 vs. 63 nm and 72 vs. 69 nm); it was speculated that the small particles had *T* = 3 geometry [34]. However, the two classes of particles could not be confirmed by cryoEM [35]. This could be because methods for in silico separation of different classes of particles occurring within the same dataset were not well-developed at that time. If the *T = 3* particles in fact package non-replicative alphaviral subgenomic RNA or cellular RNA, they could be of interest as a future alphavirus vaccine or mRNA delivery platform. The relative infectivity and immunogenicity of particles differing in triangulation number remains to be assessed. The different quasi-equivalent interactions in the *T = 3* virion ultimately highlights the structural plasticity of the alphavirus spike lattice self-assembly. We recently reported rare flat sheet and tubular lattices of hexagonal spike arrays in CHIKV-infected cells, further demonstrating the ability of spike assemblies to cover different underlying membrane geometries [24]. It can be expected that alphavirus spikes can form arrays with numerous different protein–protein interfaces due to their extensive lateral contact areas. In summary, we demonstrated that alphavirus particles can assemble using an alternate triangulation number. That an alternate assembly lattice should be possible has been proposed but not previously experimentally observed [7]. Here, *T* = 3 particles—having 180 copies each of the envelope glycoproteins and capsid protein instead of 240 copies—constituted about 1 of every 15 particles formed during cell-culture propagation of a biosafe derivative of Eastern equine encephalitis virus.

## Figures and Tables

**Table 1:** Distribution of particle morphologies in cryo-electron micrographs of four alphaviruses and one parvovirus (adeno-associated virus serotype 8, a non-pleomorphic icosahedral virus): this table shows the number of viral particles counted with each morphology.

Preparation	Normal	Larger	Smaller	Multi-Cored
EILV/EEEV	545	29	24	19
EILV/VEEV	806	37	37	9
EILV/CHIKV	579	2	2	18
VEEV	719	19	19	25
AAV8	551	3	3	0

## Data Availability

The two EILV/EEEV cryoEM maps produced in this study are available from the EM Data Resource and from the Electron Microscopy Data Bank with accession number EMD-28780.
